# Prognostic nutritional index is useful for predicting the prognosis of patients with infective endocarditis undergoing surgery: a retrospective study

**DOI:** 10.3389/fnut.2025.1685875

**Published:** 2025-10-14

**Authors:** Kadeyanmu Abulimiti, Zheng Liu, Maierhaba Dawuti, Alapati Waili, Lin Shi, Weimin Zhang

**Affiliations:** 1 Department of Cardiac Surgery, The First Affiliated Hospital of Xinjiang Medical University, Ürümqi, Xinjiang, China; 2 Department of Critical Care Medicine, The Fifth Affiliated Hospital of Xinjiang Medical University, Ürümqi, Xinjiang, China; 3 Department of Pancreatic Surgery, The First Affiliated Hospital of Xinjiang Medical University, Ürümqi, Xinjiang, China

**Keywords:** malnutrition, prognostic nutritional index (PNI), infective endocarditis, cardiac surgery, cardiopulmonary bypass (CPB)

## Abstract

**Objective:**

To investigate the predictive efficacy of preoperative prognostic nutritional index (PNI) for clinical outcomes in patients with infective endocarditis (IE) undergoing surgical treatment.

**Methods:**

A retrospective analysis was conducted on 373 IE patients who underwent cardiac valve surgery in the Department of Cardiac Surgery, the First Affiliated Hospital of Xinjiang Medical University from January 1, 2009 to December 31, 2023. According to the preoperative PNI scores, patients were divided into the malnourished group (132 cases) and the well-nourished group (241 cases). Univariate and multivariate Logistic regression analyses were used to explore the association between preoperative PNI and postoperative in-hospital mortality as well as 1-year all-cause mortality.

**Results:**

This study analyzed 373 patients who underwent surgery for IE to assess the impact of preoperative nutritional status on prognosis. According to the preoperative PNI, 35.4% (132 patients) were malnourished. Compared with the well-nourished group, the malnourished group had a lower body mass index (BMI) (20.94 vs. 22.84, *P* < 0.001) and a higher proportion of NYHA class III–IV heart function (53.79% vs. 31.95%, *P* < 0.001). Multivariate analysis revealed that a lower preoperative PNI score (OR = 0.91, 95% CI: 0.83∼0.99, *P* = 0.033) and longer cardiopulmonary bypass (CPB) time (per 10-min increase: OR = 1.15, 95% CI: 1.07∼1.24, *P* < 0.001) were independent risk factors for postoperative in-hospital mortality. Similarly, preoperative PNI score (OR = 0.95, 95% CI: 0.90–0.99, *P* = 0.040) and CPB time (per 10-min increase: OR = 1.10, 95% CI: 1.04–1.16, *P* = 0.001) were also independent predictors of 1-year all-cause mortality. ROC curve analysis showed that the predictive ability of PNI for postoperative in-hospital mortality (AUC = 0.74) was better than that for 1-year mortality (AUC = 0.61).

**Conclusion:**

Preoperative PNI score can effectively reflect the nutritional-immune status of patients with IE. It is not only an independent risk factor for predicting postoperative in-hospital mortality and 1-year all-cause mortality, but also has certain short-term predictive ability and identification value for long-term outcomes, which can provide a reference for clinical early identification of high-risk patients and formulation of nutritional intervention strategies.

## 1 Introduction

Infective endocarditis (IE) is a severe infectious disease with concerning morbidity and mortality, posing a significant threat to human health. The latest statistics indicate that the global incidence of IE is approximately 13.8 cases per 100,000 population, resulting in around 66,300 deaths annually (1). IE can lead to severe complications such as valvular damage, sepsis, septic embolism, stroke, and acute heart failure, which not only endanger patients’ lives but also increase the difficulty of treatment and nutritional consumption, thereby elevating the risk of malnutrition (2).

In recent years, studies have found that malnutrition is associated with increased mortality, higher infection risk, and prolonged hospital stays in IE patients, significantly impairing their prognosis (3, 4). Preoperative nutritional status has a notable impact on postoperative outcomes in patients undergoing cardiac surgery (5). Research by Juliana et al. (6) demonstrated that patients with heart disease who are malnourished preoperatively have a higher mortality rate than those with good nutritional status. Additionally, patients undergoing cardiac surgery are more prone to postoperative complications such as cardiac cachexia, sarcopenia, and cognitive dysfunction, accompanied by longer intensive care unit (ICU) stays, prolonged postoperative mechanical ventilation, and higher incidences of postoperative cardiovascular and infectious complications. Therefore, preoperative nutritional status is a key driver of clinical outcomes in cardiac surgery patients.

As a serology-based tool for assessing immunonutritional status, the prognostic nutritional index (PNI) has been confirmed to be significantly associated with in-hospital and long-term mortality in various cardiovascular diseases, including acute coronary syndrome and heart failure (7–10). However, research on its role in prognostic assessment of IE remains limited. Against this backdrop, this study focuses on exploring the predictive efficacy of preoperative PNI scores for clinical outcomes in IE patients undergoing surgical treatment.

## 2 Materials and methods

### 2.1 Study population

**FIGURE 1:**
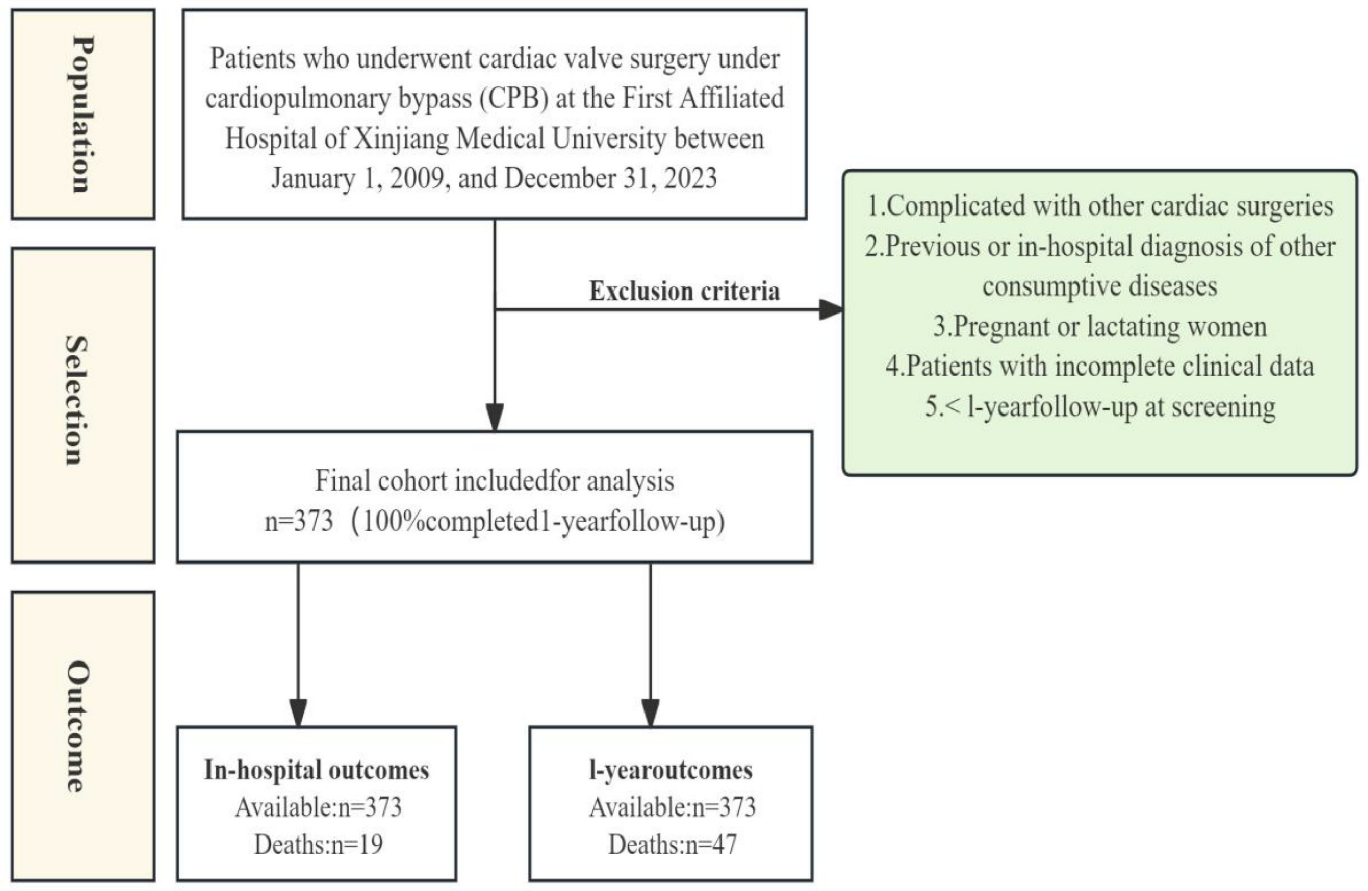
Study flow diagram.

This was a retrospective observational study. The study population included patients who were hospitalized in the Department of Cardiac Surgery, the First Affiliated Hospital of Xinjiang Medical University, from January 1, 2009, to December 31, 2023. This study has been approved by the Hospital Ethics Committee (No. K202411-32) and supported by the Open Projects of the National Key Laboratory for the Etiology and Prevention of High-Incidence Diseases in Central Asia, co-built by the Province and the Ministry Fund (No: SKLHIDCA-2023-24). These patients were clearly diagnosed with IE according to the modified Duke diagnostic criteria (11) and underwent valve surgery under cardiopulmonary bypass (CPB). Inclusion criteria: (1) Diagnosis of IE based on the modified Duke diagnostic criteria; (2) Eligibility for and receipt of cardiac surgery; (3) Adults aged ≥18 years; (4) Complete clinical data. (5) Complete 1-year follow-up data (confirmed via outpatient records and telephone interviews, with no missing information on all-cause mortality). Exclusion criteria: (1) Complicated with other cardiac surgeries (e.g., congenital heart disease, coronary atherosclerotic heart disease, Marfan syndrome, ascending aortic aneurysm, etc.); (2) Previous or in-hospital diagnosis of other consumptive diseases (e.g., malignant tumors, immunodeficiency-related diseases, hyperthyroidism, etc.); (3) Pregnant or lactating women; (4) Patients with incomplete clinical data. Additionally, to ensure that all patients ultimately included in the study cohort have complete follow-up information, those with incomplete 1-year follow-up data were excluded during the initial enrollment phase. The detailed screening process is shown in Figure 1.

### 2.2 Treatment methods

In accordance with the 2023 ESC guidelines (1), the surgery aimed to completely remove the infected foci to prevent persistent or recurrent infection, and to repair or replace the damaged valves based on the extent of valvular lesions. All patients underwent open-heart surgery with CPB to remove intracardiac vegetations and perform valve surgery. After the operation, all patients were transferred to the cardiac surgery ICU for close monitoring and treatment.

### 2.3 Data collection

Clinical data of patients were collected from the hospital’s electronic medical record system, and baseline data were entered into Excel. Baseline data included: (1) Demographic data: age, gender, BMI, etc.; (2) Symptoms and signs at admission: fever, shortness of breath, chest tightness or pain, lower extremity edema, etc.; (3) Comorbidities: diabetes, hypertension, etc.; (4) Preoperative laboratory tests: blood routine, coagulation function, liver and kidney function, etc.; (5) Surgery-related clinical data; (6) Follow-up data.

### 2.4 Malnutrition scoring systems

#### 2.4.1 Body mass index (BMI) scoring

Based on the World Health Organization (WHO) criteria, patients were classified into malnutrition (<18.5 kg/m^2^), normal weight (18.5–24.9 kg/m^2^), overweight (25.0–29.9 kg/m^2^), and obese (≥30 kg/m^2^) according to their body mass index (BMI).

#### 2.4.2 Prognostic nutritional index scoring

It mainly includes serum albumin and total peripheral blood lymphocyte count. The formula for PNI is: PNI = serum albumin (g/L) + 5 × total peripheral blood lymphocyte count (×10^9^/L) – with the following nutritional status cut-offs immediately defined: a score >38 indicates normal nutrition, scores of 35–38 indicate moderate malnutrition, and a score <35 indicates severe malnutrition (8).

### 2.5 Follow-up and grouping

All patients with infective endocarditis were followed up for 1 year through outpatient visits and telephone calls. Given that patients with incomplete follow-up data were excluded during enrollment, all 373 finally included patients completed the 1-year follow-up, achieving a 100% follow-up rate. The endpoint of follow-up was all-cause mortality. A self-designed follow-up form was used to record all-cause mortality events during follow-up. Patients were divided into the malnourished group (132 cases) and the well-nourished group (241 cases) according to preoperative PNI scores.

### 2.6 Statistical analysis

Statistical analyses were performed using SPSS version 26.0 and R version 4.4.2. Continuous variables were tested for normality using the Shapiro-Wilk test. Those conforming to a normal distribution were expressed as mean ± standard deviation and compared between groups using the independent samples *t*-test; non-normally distributed variables were expressed as median (interquartile range) and compared using the Mann-Whitney U test. Categorical variables were described as frequency (percentage) and compared using the χ^2^ test or Fisher’s exact test. Multivariable logistic regression analysis was employed to identify independent risk factors, incorporating variables with a *P*-value < 0.05 in univariate analysis and clinically relevant variables. Multicollinearity was assessed using the variance inflation factor. Receiver operating characteristic (ROC) curve analysis was conducted to evaluate predictive performance, calculating the area under the curve (AUC) with its 95% confidence interval. Differences between AUCs of different ROC curves were compared using DeLong’s test. All statistical tests were two-tailed, and a *P*-value < 0.05 was considered statistically significant.

## 3 Results

### 3.1 Baseline preoperative characteristics of patients

Among the 373 enrolled patients, the mean age was 41.9 years (range: 18–73 years), with 292 males (78.3%) and 81 females (21.7%). Regarding clinical features, weight loss of more than 5% within 3 months was the most common presentation (96 cases, 25.7%). Comorbidities and relevant medical history included 12 patients with prosthetic valves (PV, 3.2%), 20 cases of diabetes mellitus (5.4%), 53 cases of hypertension (14.2%), and 60 patients with a history of cerebrovascular accident (CVA, 16.1%). In addition, there were 16 cases of liver dysfunction (4.3%), 32 cases of renal dysfunction (8.6%), and 12 cases of electrolyte disorders (3.2%). A total of 196 patients (52.5%) had positive blood cultures. The pathogen distribution was as follows: 100 cases of *Streptococcus* infection (26.8%), 29 cases of *Staphylococcus* infection (7.8%), and notably, 10 cases of Brucellosis (2.7%). Echocardiographic findings revealed 364 patients with cardiac vegetations (97.6%), 342 patients with left-sided infective endocarditis (91.7%), 73 patients with aortic valve perforation (19.6%), and 13 patients with aortic perivalvular abscess (3.5%). Additionally, 8 cases (2.1%) had a left ventricular ejection fraction ≤40%. The distribution of surgical procedures was as follows: 148 cases (39.7%) underwent aortic valve replacement/repair, 114 cases (30.6%) underwent mitral valve replacement/repair, 3 cases (0.8%) underwent tricuspid valve replacement/repair, and 108 cases (28.9%) underwent combined valve replacement/repair.

**FIGURE 2:**
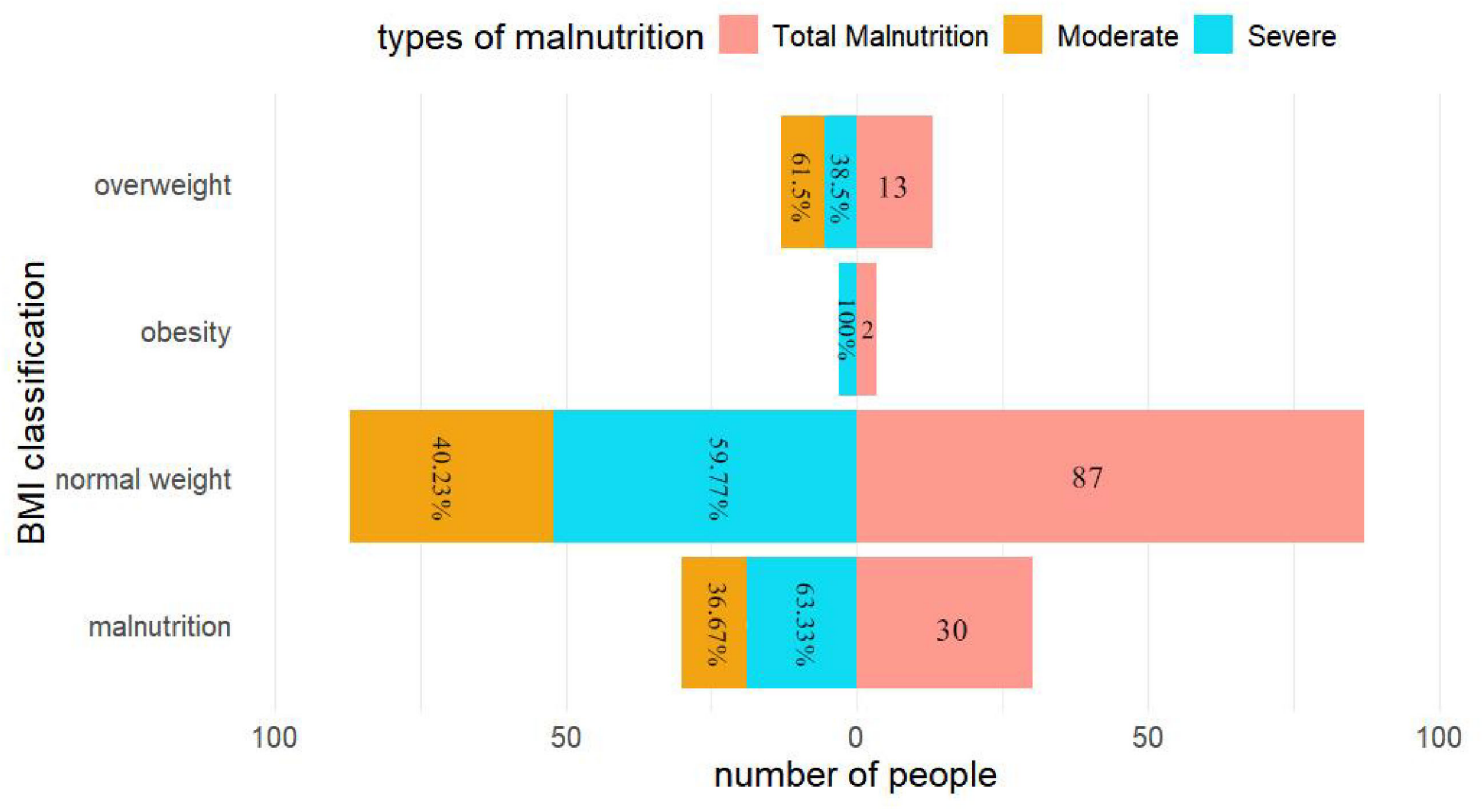
Multidimensional analysis of preoperative malnutrition in patients based on BMI and PNI.

The mean preoperative BMI of the patients was 22.2 kg/m^2^. Based on BMI classification: 58 cases (15.6%) were malnourished (<18.5 kg/m^2^), 234 cases (62.7%) had normal weight (18.5–24.9 kg/m^2^), 69 cases (18.5%) were overweight (25.0–29.9 kg/m^2^), and 12 cases (3.2%) were obese (>30 kg/m^2^). The mean preoperative PNI was 40.7. According to PNI classification, 132 cases (35.4%) were malnourished, including 54 cases of moderate malnutrition and 78 cases of severe malnutrition. Further analysis revealed that among the 132 malnourished patients identified by PNI, 87 cases (65.9%) had a normal preoperative BMI, and only 30 cases (22.7%) met the BMI criteria for malnutrition. Among the 78 cases with severe malnutrition, 52 cases (66.7%) still had a normal preoperative BMI (Figure 2 and Table 1).

**TABLE 1:** Cross-distribution of preoperative BMI categories and PNI status in patients with infective endocarditis (column %).

	Malnutrition (<18.5) (n = 58)	Normal weight (18.5–24.9) (n = 234)	Overweight (25–29.9) (n = 69)	Obese (≥30) (n = 12)
PNI malnourished (≤38)	30 (22.7%)	87 (65.9%)	13 (9.9%)	2 (1.5%)
PNI normal (>38)	28 (11.6%)	147 (61.0%)	56 (23.2%)	10 (4.2%)

### 3.2 Comparison of preoperative clinical characteristics

Between IE Malnourished Group and Well-Nourished Group According to the preoperative PNI scoring criteria, patients were divided into the malnourished group (132 cases, 35.4%) and the well-nourished group (241 cases, 64.6%). The age distribution of the two groups was relatively similar. Comparison of clinical data (Table 2) showed that, compared with the well-nourished group, the malnourished group was significantly associated with lower BMI (20.94 ± 3.38 vs. 22.84 ± 3.80, *P* < 0.001) and had a higher proportion of cardiac insufficiency with New York Heart Association (NYHA) Classification III-IV (53.79% vs. 31.95%, *P* < 0.001). Comparison of laboratory test results (Table 3) revealed that the levels of B-Type Natriuretic Peptide, procalcitonin, and Neutrophil-to-Lymphocyte Ratio (NLR) in the malnourished group were significantly higher than those in the well-nourished group (all *P* < 0.01); while the levels of creatine kinase, total cholesterol, serum albumin, hemoglobin, and platelet count were significantly lower (all *P* < 0.05).

**TABLE 2:** Comparison of clinical data between the well-nourished group and the malnourished group.

Variables	The well-nourished group N = 241^1^	The malnourished group N = 132^1^	*P*-value^2^
Age	41.79 (12.15)	42.15 (11.64)	0.638
Gender			0.161
Female	47 (19.50%)	34 (25.76%)	
Male	194 (80.50%)	98 (74.24%)	
Body mass index	22.84 (3.80)	20.94 (3.38)	<0.001
Hypertension	33 (13.69%)	20 (15.15%)	0.700
Diabetes	13 (5.39%)	7 (5.30%)	0.970
History of CVA	36 (14.94%)	24 (18.18%)	0.415
Liver dysfunction	8 (3.32%)	8 (6.06%)	0.212
Renal insufficiency	18 (7.47%)	14 (10.61%)	0.301
NYHA classification III-IV	77 (31.95%)	71 (53.79%)	<0.001
Prosthetic valve, PV	5 (2.07%)	7 (5.30%)	0.167
Left-sided infective endocarditis	220 (91.29%)	122 (92.42%)	0.703
**Microbiological characteristics**			
*Streptococcus*	67 (27.80%)	33 (25.00%)	0.559
*Staphylococcus*	11 (4.56%)	18 (13.64%)	0.002
*Enterococcus*	4 (1.66%)	4 (3.03%)	0.617
Brucellosis	8 (3.32%)	2 (1.52%)	0.486
**Echocardiographic characteristics**			
Vegetation	235 (97.51%)	129 (97.73%)	0.967
Aortic valve perforation	47 (19.50%)	26 (19.70%)	0.964
Aortic perivalvular abscess	6 (2.49%)	7 (5.30%)	0.262

^1^Mean (SD); *n* (%).

^2^Wilcoxon rank sum test; Pearson’s Chi-squared test; Fisher’s exact test.

**TABLE 3:** Comparison of laboratory test results between the well-nourished group and the malnourished group.

Variables	The well-nourished group N = 241^1^	The malnourished group N = 132^1^	*P*-value^2^
Creatine kinase, IU/L	72.65 (154.39)	50.77 (72.80)	<0.001
B-type natriuretic peptide, pg/ml	2,774.25 (4,435.30)	5,217.35 (7,244.94)	<0.001
Total cholesterol, mmol/L	3.53 (0.91)	2.98 (0.93)	<0.001
Serum albumin, g/L	35.44 (4.70)	27.15 (3.50)	<0.001
White blood cell count, ×10^9^/L	8.53 (3.62)	8.40 (3.84)	0.652
Lymphocyte count, ×10^9^/L	1.82 (0.73)	1.26 (0.48)	<0.001
Neutrophil count, ×10^9^/L	5.87 (3.25)	6.39 (3.67)	0.082
Hemoglobin, g/L	119.13 (21.03)	96.56 (18.42)	<0.001
Platelet count, ×10^9^/L	237.23 (87.03)	205.13 (88.23)	0.002
C-reactive protein, mg/L	31.69 (39.91)	44.90 (39.20)	<0.001
Procalcitonin, ng/ml	0.50 (2.37)	0.91 (3.39)	<0.001
Neutrophil-to-lymphocyte ratio (NLR)	4.22 (5.74)	5.78 (4.32)	<0.001

^1^Mean (SD); *n* (%).

^2^Wilcoxon rank sum test; Pearson’s Chi-squared test; Fisher’s exact test.

### 3.3 Model diagnostics: collinearity (VIF)

To assess multicollinearity, variance inflation factors [VIF = 1/(1−R^2^)] were computed using ordinary least squares. All covariates in both multivariable models had VIFs close to 1 and <2 (approximate range 1.02∼1.09), indicating no meaningful multicollinearity (Supplementary Tables 1, 2). Because serum albumin is a component of PNI, albumin was not entered alongside PNI to avoid structural collinearity.

### 3.4 Analysis of risk factors for postoperative in-hospital mortality in IE patients

In this study, 19 cases (5.1%) of postoperative in-hospital mortality occurred among IE patients. Univariate logistic regression analysis (Table 4) showed that NYHA class III–IV (OR = 3.51, 95% CI: 1.35∼10.21, *P* = 0.013), preoperative PNI score (OR = 0.87, 95% CI: 0.80∼0.94, *P* < 0.001), and CPB time modeled per 10-min increment were significantly associated with postoperative in-hospital mortality (OR = 1.10, 95% CI: 1.01∼1.34, *P* < 0.001). After including variables with *P* < 0.05 from the above univariate analysis into the multivariable logistic regression (Table 5) and modeling CPB time per 10 min, preoperative PNI score (OR = 0.91, 95% CI: 0.83∼0.99, *P* = 0.033) and CPB time (per 10 min: OR = 1.15, 95% CI: 1.07∼1.24, *P* < 0.001) remained independently associated with postoperative in-hospital mortality in IE patients.

**TABLE 4:** Univariate analysis of risk factors for in-hospital mortality in IE patients undergoing surgery.

Variables	Any (n = 373)	Univariate analysis	
		OR (95% CI)	*P*
Age	373 (100.0%)	1.03 (0.99∼1.07)	0.167
Female	81 (21.7%)	1.72 (0.59∼4.50)	0.290
Preoperative NYHA classification III-IV	148 (40.0%)	3.51 (1.35∼10.21)	0.013
Preoperative renal insufficiency	32 (8.6%)	1.27 (0.20∼4.73)	0.756
Preoperative BMI score	373 (100.0%)	0.98 (0.86∼1.11)	0.752
Preoperative PNI score	373 (100.0%)	0.87 (0.80∼0.94)	<0.001
Cardiopulmonary bypass time	373 (100.0%)	1.10 (1.01∼1.34)	<0.001

Cardiopulmonary bypass time is modeled per 10-min increment; OR indicates the change in odds per additional 10 min of CPB time.

**TABLE 5:** Multivariate analysis of risk factors for in-hospital mortality in IE patients undergoing surgery.

Variables	Any (n = 373)	Multivariate analysis	
		OR (95% CI)	*P*
Preoperative NYHA classification III-IV	148 (40.0%)	1.74 (0.57∼5.70)	0.335
Preoperative PNI score	373 (100.0%)	0.91 (0.83∼0.99)	0.033
Cardiopulmonary bypass time	373 (100.0%)	1.15 (1.07∼1.24)	<0.001

Cardiopulmonary bypass time is modeled per 10-min increment; OR indicates the change in odds per additional 10 min of CPB time.

### 3.5 Analysis of risk factors for postoperative 1-year all-cause mortality in IE patients

A total of 47 cases (12.6%) of 1-year all-cause mortality occurred after surgery in IE patients. Univariate logistic regression (Table 6) showed that age (OR = 1.03, 95% CI: 1.01∼1.06, *P* = 0.029), preoperative PNI score (OR = 0.93, 95% CI: 0.89∼0.98, *P* = 0.005), and CPB time modeled per 10-min increment were significantly associated with 1-year all-cause mortality (OR = 1.11, 95% CI: 1.10∼1.18, *P* < 0.001). In the multivariable logistic regression (Table 7) with CPB time modeled per 10 min, preoperative PNI score (OR = 0.95, 95% CI: 0.90∼0.99, *P* = 0.040) and CPB time (per 10 min: OR = 1.10, 95% CI: 1.04∼1.16, *P* = 0.001) remained independent predictors of 1-year all-cause mortality, whereas age was no longer statistically significant (*P* = 0.118).

**TABLE 6:** Univariate analysis of risk factors for 1-year all-cause mortality in IE patients undergoing surgery.

Variables	Any (n = 373)	Univariate analysis	
		OR (95% CI)	*P*
Age	373 (100.0%)	1.03 (1.01∼1.06)	0.029
Female	81 (21.7%)	1.12 (0.52∼2.24)	0.764
Preoperative NYHA classification III-IV	148 (40.0%)	1.54 (0.83∼2.85)	0.167
Preoperative renal insufficiency	32 (8.6%)	1.69 (0.60∼4.11)	0.278
Preoperative BMI score	373 (100.0%)	0.98 (0.90∼1.06)	0.548
Preoperative PNI score	373 (100.0%)	0.93 (0.89∼0.98)	0.005
Cardiopulmonary bypass time	373 (100.0%)	1.11 (1.10∼1.18)	<0.001

Cardiopulmonary bypass time is modeled per 10-min increment; OR indicates the change in odds per additional 10 min of CPB time.

**TABLE 7:** Multivariate analysis of risk factors for 1-year all-cause mortality in IE patients undergoing surgery.

Variables	Any (n = 373)	Multivariate analysis	
		OR (95% CI)	*P*
Age	373 (100.0%)	1.02 (0.99∼1.05)	0.118
Preoperative PNI score	373 (100.0%)	0.95 (0.90∼0.99)	0.040
Cardiopulmonary bypass time	373 (100.0%)	1.10 (1.04∼1.16)	0.001

Cardiopulmonary bypass time is modeled per 10-min increment; OR indicates the change in odds per additional 10 min of CPB time.

### 3.6 Functional form and linearity

For in-hospital mortality, PNI showed significant non-linearity (RCS, P_non–linearity_−0.0277), while CPB time did not (*P* = 0.087). For 1-year mortality, CPB time exhibited clear non-linearity (*P* = 0.001), whereas PNI did not (*P* = 0.152). Given event counts and parsimony, we retained linear terms in primary models; spline curves are presented as sensitivity analyses (Supplementary Figures 1, 2). For clinical interpretability, we further report OR per 5-point decrease in PNI from the multivariable models: In-hospital mortality: OR per 1-point increase = 0.91 (95% CI: 0.83∼0.99), OR per 5-point decrease = 1.60 (95% CI: 1.05∼2.54). 1-year mortality: OR per 1-point increase = 0.95 (95% CI: 0.90–0.99), OR per 5-point decrease = 1.29 (95% CI: 1.05∼1.69).

### 3.7 Absolute risks and adjusted ORs by PNI strata/quartiles

To enhance clinical interpretability, the PNI was categorized into three strata: severe malnutrition (PNI < 35), moderate malnutrition (PNI 35–38), and normal nutrition (PNI > 38). Absolute risks with 95% confidence intervals (CIs), calculated using the Clopper–Pearson method, are reported for each stratum.

For in-hospital mortality (Table 8), after adjusting for CPB time (per 10-min increment) and NYHA class III–IV, the absolute risk was 2.5% (95% CI: 0.9–5.3) in the normal PNI group, 7.4% (95% CI: 2.1–17.9) in the moderate malnutrition group, and 11.5% (95% CI: 5.4–20.8) in the severe malnutrition group. Compared to the normal PNI group, the adjusted odds ratios were 2.54 (95% CI: 0.66–9.81, *P* = 0.178) for moderate malnutrition and 2.14 (95% CI: 0.61–7.53, *P* = 0.236) for severe malnutrition.

**TABLE 8:** In-hospital mortality: absolute risks and adjusted ORs by PNI strata.

PNI stratum	Absolute risk% (95% CI)	Adjusted OR (95% CI)	*P*
Normal > 38	2.5% (0.9–5.3)	1.00 (reference)	—
Moderate 35–38	7.4% (2.1–17.9)	2.54 (0.66–9.81)	0.178
Severe < 35	11.5% (5.4–20.8)	2.14 (0.61–7.53)	0.236

Risks are event rates with Clopper–Pearson 95% CIs. Multivariable model adjusted for CPB time (per 10 min) and NYHA class III∼IV.

For 1-year all-cause mortality (Table 9), after adjusting for age and CPB time (per 10-min increment), the absolute risk was 9.5% (95% CI: 6.1–14.0) in the normal PNI group, 13.0% (95% CI: 5.4–24.9) in the moderate malnutrition group, and 21.8% (95% CI: 13.2–32.6) in the severe malnutrition group. The adjusted odds ratios were 1.33 (95% CI: 0.53–3.34, *P* = 0.540) for moderate malnutrition and 1.98 (95% CI: 0.93–4.18, *P* = 0.075) for severe malnutrition, using the normal PNI group as reference. Although the severe malnutrition group showed a trend toward higher mortality risk, the adjusted OR were not statistically significant (*P* > 0.05).

**TABLE 9:** 1-year all-cause mortality: absolute risks and adjusted ORs by PNI strata.

PNI stratum	Absolute risk% (95% CI)	Adjusted OR (95% CI)	*P*
Normal > 38	9.5% (6.1–14.0)	1.00 (reference)	–
Moderate 35–38	13.0% (5.4–24.9)	1.33 (0.53–3.34)	0.540
Severe < 35	21.8% (13.2–32.6)	1.98 (0.93–4.18)	0.075

Risks are event rates with Clopper-Pearson 95% CIs. Multivariable model adjusted for age and CPB time (per 10 min).

### 3.8 Predictive efficacy of PNI for prognosis in patients with infective endocarditis by ROC curve

**FIGURE 3:**
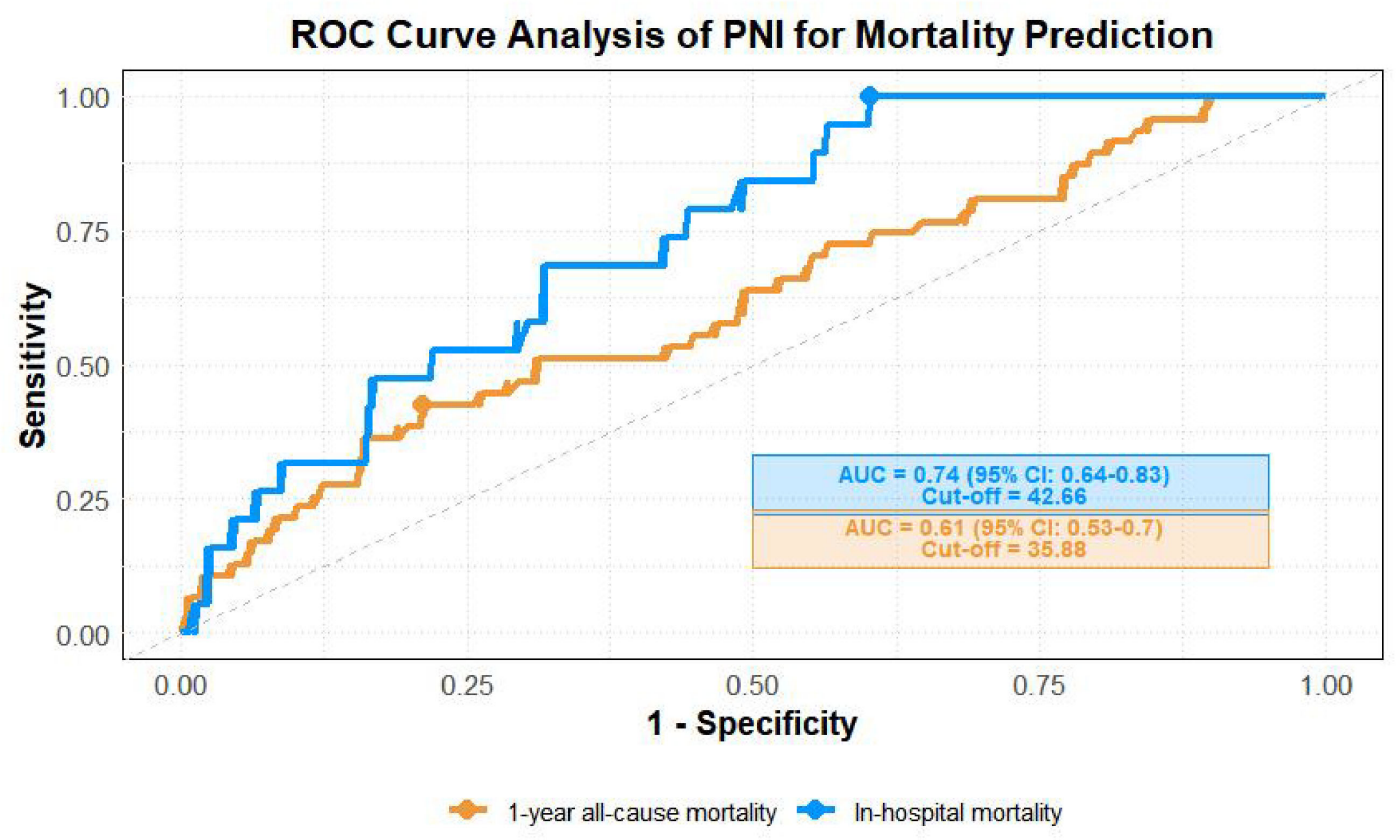
Predictive efficacy of PNI for in-hospital death and 1-year all-cause death in patients with infective endocarditis.

Results of ROC curve analysis showed that the area under the curve (AUC) of PNI for predicting postoperative in-hospital death in patients with infective endocarditis was 0.74 (95% CI: 0.64–0.83), and the AUC for predicting 1-year all-cause death was 0.61 (95% CI: 0.53∼0.70) (Figure 3).

## 4 Discussion

Despite the proliferation of research on IE, little is known about the relationship between the PNI and IE. Based on our findings, three key observations can be made. First, IE patients classified as malnourished or well-nourished by PNI criteria exhibit distinct clinical characteristics. The malnourished group shows lower BMI and a higher proportion of NYHA Classification III-IV (53.79% vs. 31.95%). They also have significantly elevated inflammatory markers (e.g., C-reactive protein, procalcitonin) and markedly reduced nutrition-related indicators (e.g., serum albumin, lymphocytes). These findings suggest that preoperative PNI can effectively distinguish the nutritional-inflammatory status and degree of cardiac function impairment in IE patients. Second, the preoperative PNI score is an independent risk factor for in-hospital mortality and 1-year all-cause mortality in IE patients undergoing surgery. A lower score indicates higher risk. This score demonstrates predictive efficacy for both short-term and long-term prognosis. Prolonged CPB time is also an independent risk factor, suggesting that surgical trauma and nutritional-immune status jointly influence prognosis.

These results further supplement existing research. Several surgical risk scores (e.g., EuroSCORE II, ACC/AHA risk models) include age, heart failure, renal failure, microbial type, and local complications (12, 13). However, their application is limited by heterogeneity in IE manifestations and variable disease progression. Kahraman et al. (14) first reported that PNI is associated with in-hospital mortality in IE inpatients, providing a basis for this study. Our analysis confirmed that preoperative PNI score is an independent risk factor for 1-year all-cause mortality in surgically treated IE patients (OR = 0.95, 95% CI: 0.90∼0.99, *P* = 0.040). Limited evidence has addressed the long-term prognostic value of PNI in this population. Kahraman et al. reported an association between PNI and in-hospital mortality, but did not explore 1-year outcomes. Our findings extend their work by confirming PNI’s independent predictive role for long-term mortality. Meanwhile, its independent predictive value for in-hospital mortality was also verified (OR = 0.91, 95% CI: 0.83∼0.99, *P* = 0.03). This result is consistent with Cheng et al. (15) In very elderly patients with non-valvular atrial fibrillation, moderate to severe malnutrition assessed by PNI was an independent predictor of composite events (thromboembolism + all-cause death) (HR = 3.37, 95% CI: 1.90∼6.00, *P* < 0.001). This confirms the universal significance of PNI as a prognostic indicator in cardiovascular diseases. PNI directly reflects the patient’s underlying status rather than acting through complications. Patients with abnormal preoperative PNI are more likely to develop uncontrolled infection and heart failure due to systemic imbalance. This effect is evident in both short-term in-hospital outcomes and long-term prognosis. Thus, PNI serves as a new independent predictor for long-term risk assessment in IE patients. Similar conclusions were reported by Chen et al. (16, 17), who showed that PNI predicts both short-term and long-term prognosis in heart disease patients.

Notably, in the multivariate analysis of in-hospital mortality, NYHA class lost significance after adjustment (*P* = 0.335). PNI, however, remained independently associated with prognosis. This suggests that PNI’s effect is not dependent on NYHA classification and highlights its unique role as a supplementary risk indicator. ROC curve analysis confirmed its predictive value. The AUC for in-hospital mortality was 0.74 (95% CI: 0.64∼0.83), indicating moderate predictive efficacy. The AUC for 1-year mortality was 0.61 (95% CI: 0.53∼0.70), showing modest identification value. This aligns with the trend of PNI’s C-index (0.77, 95% CI: 0.71∼0.82) in cardiac valve surgery patients reported by Cho et al. (18) The weaker performance for long-term outcomes reflects the complexity of prognosis, influenced by postoperative interventions and disease progression. Nonetheless, PNI retains unique value as a preoperative marker. It is based on quantitative serological data and does not require complex indicators. PNI provides objective evidence of the link between nutritional-immune status and prognosis. It is a simple, independent tool that supports clinical decision-making, risk stratification, and outcome prediction in cardiovascular disease (19–21).

A key phenomenon emerged in our study. Among the 132 malnourished patients identified by PNI, the majority (65.9%, 87 cases) had normal BMI. In addition, 11.4% (15 cases) were overweight or obese. These data show that normal BMI does not always equal good nutrition, and obesity may coexist with malnutrition. Compared with BMI, which only categorizes body shape, PNI better captures the essence of nutritional-immune status. It helps avoid delays in clinical decisions caused by misjudging nutrition based on body weight.

The predictive advantage of PNI comes from its integration of albumin and lymphocytes. Albumin and lymphocytes are both crucial in IE progression. In this study, serum albumin in the malnourished group was markedly lower than in the well-nourished group (27.15 vs. 35.44 g/L, *P* < 0.001). This is consistent with findings that hypoalbuminemia is an independent risk factor for early postoperative mortality in IE (22). Hypoalbuminemia not only reflects malnutrition but also reduces immunoglobulin synthesis, impairs cellular immunity, and weakens pathogen clearance. This creates a vicious cycle of “malnutrition–immunosuppression–disease progression” (23). Similarly, lymphocyte counts were significantly lower in the malnourished group (1.26 vs. 1.82 × 10^9^/L, *P* < 0.001). Low lymphocyte count is closely linked to severity and mortality of infectious diseases (24, 25). Zhang et al. (26) also showed that a total lymphocyte count <1500/mm^3^ is strongly associated with malnutrition. Thus, low lymphocytes in IE patients reflect both immune dysfunction and nutritional deficiency, increasing the risk of poor outcomes. By integrating these two markers, PNI captures the “nutrition–inflammation–immunity” interaction, which explains its superior predictive value.

Prognostic nutritional index has direct clinical implications. Patients with low PNI should receive individualized nutritional intervention via multidisciplinary collaboration (cardiac surgery and nutrition teams). Albumin and high-quality protein should be supplemented to correct hypoalbuminemia, along with trace elements such as zinc and selenium to enhance lymphocyte function (27). At the same time, careful preoperative preparation can shorten CPB time, an independent risk factor identified in this study, thereby reducing risks of organ hypoperfusion and bleeding (28). Dynamic monitoring of PNI with cardiac and inflammatory indicators may also help detect uncontrolled infection or heart failure early. The mean age of our surgical IE cohort was 42 years, lower than the 60–70 years typically reported in Western IE cohorts (29–31). This likely reflects regional differences in case mix and etiology. In high-income countries, population aging and prosthetic/device-related IE shift the age distribution upward. In contrast, native-valve disease and streptococcal infections remain more common in some regions (32). Therefore, our findings are most generalizable to younger surgical IE populations. Extrapolation to older or device-related cases should be done with caution and ideally validated externally.

To enhance comparability, we reported key descriptors: native vs. prosthetic valve IE, left- vs. right-sided involvement, blood-culture positivity, pathogen spectrum, embolic events, and vegetation/abscess characteristics. Age was adjusted in multivariable models (33, 34). For clinical communication, we prespecified PNI categories: >38 (normal), 35–38 (moderate malnutrition), and <35 (severe malnutrition). There is no universally accepted cut-off. Kahraman et al. reported a cut-off of ≈35.6 for IE (AUC 0.691), supporting our use of the lower range. In other cardiac surgical cohorts, higher thresholds have been reported–for example, ≈46.1 in adult cardiac surgery with CPB (35) and ≈43.4–48.3 in TAVR cohorts (36, 37). This reflects population- and procedure-dependence. We therefore analyzed PNI both categorically and continuously.

Our 1-year mortality model’s AUC of 0.61 indicates modest discrimination. PNI alone has limited utility for individual risk stratification and is better as an auxiliary signal. This is consistent with recent IE studies, where single markers show only moderate accuracy, while multivariable models integrating clinical, microbiological, imaging, and perioperative factors perform better (38, 39). From a nutritional assessment perspective, PNI is complementary to indices such as GNRI and CONUT. Their relative performance varies across populations. In cardiac surgery and structural-heart cohorts, malnutrition is consistently linked with poor outcomes. Recent meta-analyses suggest GNRI may perform as well as, or even better than, PNI for short-term mortality. PNI, however, provides a simple immune-nutrition signal. Combined or comparative use of metrics is preferable to reliance on one index (40, 41). Dynamic assessment may also improve prediction. Perioperative changes in PNI have been associated with outcomes, suggesting it should be considered a time-varying risk indicator. In practice, integrating PNI with clinical factors (age, NYHA class, CPB time), microbiological/echocardiographic data, and other nutrition scores is more realistic. Based on evidence, we position PNI as an adjunct to established risk models rather than a stand-alone tool.

## 5 Limitations

This study has several limitations. First, its single-center, retrospective design, combined with a limited sample size and a highly homogeneous population, may introduce selection bias. Second, it should be noted that the mean age of the IE population in this cardiac surgery cohort is relatively young. Therefore, we explicitly caution against overgeneralizing the conclusions to elderly IE populations or those with a high incidence of device-related IE, to avoid misapplication of the findings due to differences in population characteristics. Furthermore, the PNI, as a composite parameter derived from serum albumin and lymphocyte count–both of which are influenced by nutritional status and systemic inflammation–cannot fully distinguish the independent effects of nutritional deficits from those of inflammatory responses. Thus, the PNI should be interpreted primarily as a comprehensive inflammatory-nutritional risk marker rather than a pure nutritional indicator. Third, nutritional status was assessed only at admission without capturing dynamic changes during hospitalization. Fourth, the validation of the PNI-based predictive model focused primarily on discrimination, while comprehensive assessments of calibration and clinical utility remain lacking. Future prospective studies with larger sample sizes and external validation cohorts are warranted. These should incorporate more specific nutritional indicators and employ tools such as decision curve analysis to evaluate clinical net benefit, thereby facilitating the translation of such models into clinical practice.

## Data Availability

The raw data supporting the conclusions of this article will be made available by the authors, without undue reservation.
