# Correction: Prognostic nutritional index is useful for predicting the prognosis of patients with infective endocarditis undergoing surgery: a retrospective study

**DOI:** 10.3389/fnut.2025.1736985

**Published:** 2025-11-19

**Authors:** Kadeyanmu Abulimiti, Zheng Liu, Maierhaba Dawuti, Alapati Waili, Lin Shi, Weimin Zhang

**Affiliations:** 1 Department of Cardiac Surgery, The First Affiliated Hospital of Xinjiang Medical University, Ürümqi, Xinjiang, China; 2 Department of Critical Care Medicine, The Fifth Affiliated Hospital of Xinjiang Medical University, Ürümqi, Xinjiang, China; 3 Department of Pancreatic Surgery, The First Affiliated Hospital of Xinjiang Medical University, Ürümqi, Xinjiang, China

**Keywords:** malnutrition, prognostic nutritional index (PNI), infective endocarditis, cardiac surgery, cardiopulmonary bypass (CPB)

Correction content:

1.For authors Kadeyanmu Abulimiti, Zheng Liu, Lin Shi, Weimin Zhang:

Original incorrect content: “Department of Cardiac Surgery, First Affiliated Hospital of Xinjiang Medical University, Ürümqi, China” (missing “The”, no “Xinjiang”)

Corrected content: “Department of Cardiac Surgery, The First Affiliated Hospital of Xinjiang Medical University, Ürümqi, Xinjiang, China”

2.For author Maierhaba Dawuti:

Original incorrect content: “Department of Critical Care Medicine, Fifth Affiliated Hospital of Xinjiang Medical University, Ürümqi, China” (missing “The”, no “Xinjiang”)

Corrected content: “Department of Critical Care Medicine, The Fifth Affiliated Hospital of Xinjiang Medical University, Ürümqi, Xinjiang, China”

3.For author Alapati Waili:

Original incorrect content: “Department of Pancreatic Surgery, First Affiliated Hospital of Xinjiang Medical University, Ürümqi, China” (missing “The”, no “Xinjiang”)

Corrected content: “Department of Pancreatic Surgery, The First Affiliated Hospital of Xinjiang Medical University, Ürümqi, Xinjiang, China”

The original version of this article has been updated.

