# Supplementary material for: Prognostic nutritional index is useful for predicting the prognosis of patients with infective endocarditis undergoing surgery: a retrospective study
Source: Front Nutr. 2025 Oct 14;12:1685875. doi: 10.3389/fnut.2025.1685875 (PMC12558796; doi:10.3389/fnut.2025.1685875)
Supplement: Supplementary file 1 [file Table_1.docx]

**Supplementary Material**

Supplementary Table 1. VIFs for covariates (In-hospital mortality model)

| Model | Predictor | R^2^ | VIF = 1/(1−R^2^) |
| --- | --- | --- | --- |
| In-hospital mortality | Preoperative PNI Score | 0.058 | 1.06 |
| In-hospital mortality | CPB_per10 | 0.056 | 1.06 |
| In-hospital mortality | Preoperative NYHA Classification III-IV | 0.085 | 1.09 |

Supplementary Table 2. VIFs for covariates (1-year mortality model)

| Model | Predictor | R^2^ | VIF = 1/(1−R^2^) |
| --- | --- | --- | --- |
| 1-year mortality | Age | 0.027 | 1.03 |
| 1-year mortality | Preoperative PNI Score | 0.021 | 1.02 |
| 1-year mortality | CPB per10 | 0.042 | 1.04 |


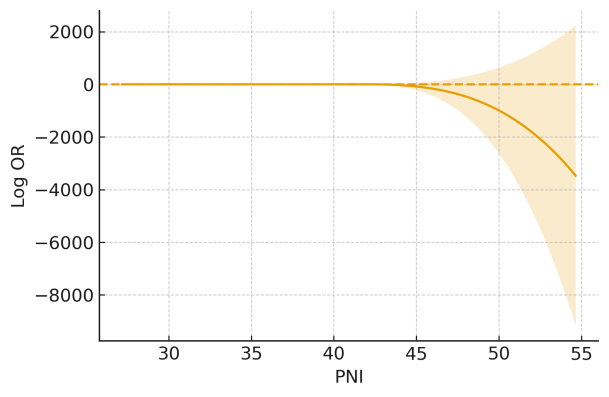

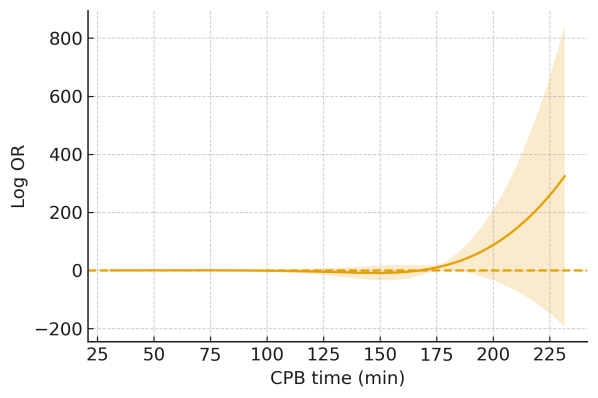


A B

Supplementary Figure 1 In-hospital mortality

A: PNI RCS :Monotonically decreasing curve; wider CI at low PNI range.B: CPB RCS :Risk increases with longer CPB; mild curvature in mid-to-high range.


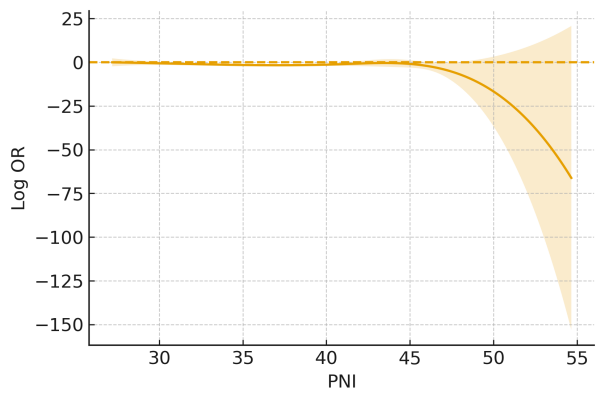

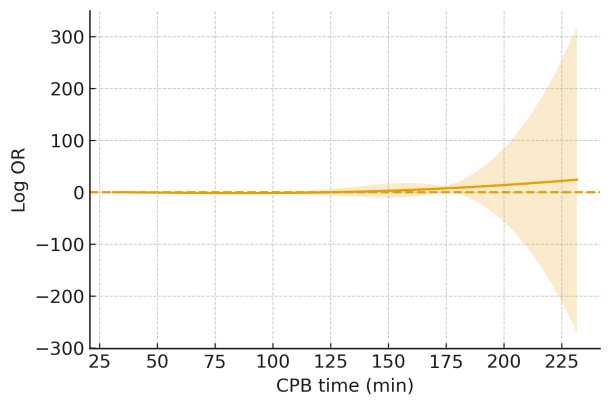


A B

Supplementary Figure 2 1-year mortality

A: PNI RCS :Approximately linear decline.B: CPB RCSClear nonlinearity: slope steepens beyond the median; risk accelerates at longer CPB .
